# High Expression of MUC5AC, MUC5B, and Layilin Plays an Essential Role in Prediction in the Development of Plastic Bronchitis Caused by MPP

**DOI:** 10.3389/fmicb.2022.911228

**Published:** 2022-06-13

**Authors:** Yu Ma, Yeqi Gu, Xinxing Zhang, Wenjing Gu, Ting Wang, Huiming Sun, Yinfang Dai, Yongdong Yan, Yuqing Wang, Meijuan Wang, Huiquan Sun, Chuangli Hao, Liping Fan, Zhengrong Chen

**Affiliations:** ^1^Department of Respiratory Medicine, Children's Hospital of Soochow University, Suzhou, China; ^2^Department of Pediatrics, Changzhou Wujin People's Hospital, Changzhou, China

**Keywords:** *Mycoplasma pneumoniae* pneumonia (MPP), plastic bronchitis (PB), MUC5AC, MUC5B, layilin

## Abstract

Plastic bronchitis (PB) is a rare respiratory condition which can result in severe respiratory complications such as respiratory failure and death. *Mycoplasma pneumoniae* infection is a main etiology cause of plastic bronchitis. However, the pathogenesis of plastic bronchitis complicated by *Mycoplasma pneumoniae* pneumonia (MPP) has not yet been fully elucidated. Our article aims to explore biomarkers for early prediction of MPP cases complicated with plastic bronchitis. We utilized a protein chip to screen for significantly different proteins among the groups of healthy, general *Mycoplasma pneumoniae* pneumonia (GMPP) and refractory *Mycoplasma pneumoniae* pneumonia (RMPP) patients, where layilin exhibited a potent change across biology information technology. Next, we demonstrated the high expression of MUC5AC, MUC5B, and layilin in bronchoalveolar lavage fluid (BALF) of MPP cases complicated with plastic bronchitis. Further study suggested that the level of layilin had a positive correlation with both MUC5AC and MUC5B. A receiver operating characteristic (ROC) analysis was performed to assess the diagnostic values of MUC5AC, MUC5B, and layilin in MPP cases with PB. Data show that the three indicators have similar diagnostic ability for MPP children with plastic bronchitis. Then, we used different concentrations of community-acquired respiratory distress syndrome (CARDS) toxin or lipid-associated membrane proteins (LAMPs) to simulate an *in vitro* experiment. The *in vitro* assay revealed that CARDS toxin or LAMPs induced A549 cells to secrete MUC5AC, MUC5B, layilin, and proinflammatory factors. These findings suggest that MUC5AC, MUC5B, and layilin are correlated with MPP. The high expression of MUC5AC, MUC5B, and layilin play an essential role in prediction in the development of plastic bronchitis caused by MPP. The high expression of MUC5AC, MUC5B, and layilin may be relevant to the severity of illness.

## Introduction

*Mycoplasma pneumoniae* (*M. pneumoniae*) is an important cause of respiratory tract infections in children as well as adults which can induce both upper and lower respiratory infections and occur both endemically and epidemically worldwide (Waites et al., 2017). While *Mycoplasma pneumoniae* is one of the most common pathogens of community-acquired pneumonia in children, in general, *Mycoplasma pneumoniae* pneumonia (MPP) is considered as a benign and self-limiting disease (Chen et al., 2015; Jain et al., 2015). Nevertheless, because of drug-resistant strains, some children infected with MP who are treated with macrolide antibiotics still progressed to refractory *Mycoplasma pneumoniae* pneumonia (RMPP) (Zhou et al., 2015; Zhu et al., 2021). Studies have shown that if flexible bronchoscopy is undertaken as early as possible, the symptoms of the children who were diagnosed with MPP or RMPP can be alleviated more quickly and the disease can be controlled (Shah et al., 2008; Efrati et al., 2009).

Plastic bronchitis (PB) is a disease characterized by bronchial casts which can lead to pulmonary necrosis and pleural effusion that are difficult to treat and costly (Rubin, 2016). The common causes of PB are pathogen infections. Previous studies have demonstrated that influenza viruses (A and B) are common pathogen causes of PB. At present, there are some domestic clinical reports on MP plastic bronchitis (Huang et al., 2019; Wang et al., 2020). However, the pathogenesis of plastic bronchitis complicated by MPP has not yet been fully elucidated.

It has been reported that the casts of PB often contain an abundance of mucin in normal mucus, which are linearly linked (Rubin et al., 2014). Over-secretion and a clearance disorder of mucus are important pathogenic mechanisms. Hao et al. (2014) demonstrated that MP induced the expression of mucins MUC5AC and MUC5B by activating the STAT6-STAT3 and epidermal growth factor receptor (EGFR) signal pathways, which in turn downregulated FOXA2. The level of MUC5AC and MUC5B has been found increased in respiratory diseases like asthma and COPD (Caramori et al., 2009; Wu et al., 2017). The high secretion of mucus is relevant to pathogenesis of MPP complicated with plastic bronchitis. All this basic research suggested that MUC5AC and MUC5B may play an important role in MPP complicated with plastic bronchitis.

Inflammation and lymphatic anomalies are important pathogenic mechanisms of plastic bronchitis. Layilin as a HA receptor has some similarities to CD44 which plays a role in the pathogenesis of mucus hypersecretion (Yu et al., 2011). Bronchial cast expectoration through bronchopulmonary lymphatics with fistula formation into the airways is a rare clinical symptom in patients with PB (Orliaguet et al., 2002). Studies showed that layilin is related to lymphatic metastasis, adhesion, migration, and invasion in lung cancer (Pan et al., 2019). However, the role of layilin in MPP complicated with plastic bronchitis is still unknown. And the relationship between layilin and MUC5AC and MUC5B has not been reported.

Therefore, this study aimed to investigate the clinical significance of MUC5AC, MUC5B, and layilin in bronchoalveolar lavage fluid (BALF) and explored predicting factors of MPP complicated with plastic bronchitis in children. In addition, we explored whether the potential mechanism of layilin upregulation was associated with MUC5AC and MUC5B.

## Materials and Methods

### Patients and Study Design

From June 2019 to October 2020, cases with MPP from Children's Hospital of Soochow University confirmed by MP-DNA-PCR > 1.0 × 10^4^ copies/ml in nasopharyngeal aspirates and BALF or MP-IgM ≥ 1.1COL or IgG titers four-fold or greater increase in serum were enrolled (*N* = 70). All patients were from 8 month to 8 years old who had fever, cough, tachypnea, chest retractions, abnormal auscultatory findings, and radiologic evidence of CAP and underwent flexible fiber optic bronchoscopy. Or the disease progression showed signs of obstruction, which suspected airway mucosal damage and airway secretions were abundant, mucus plugs was formed, and suspect developed in Bronchiolitis obliterans (An et al., 2011; Zhang et al., 2014; Yan et al., 2017). To exclude co-infection, seven respiratory virus antigen tests (influenza A and B; parainfluenza 1, 2, and 3; respiratory syncytial virus; and adenovirus) were conducted, of which HBoV, HRV, Hmpv, and CP were negative. Sputum, NPA, and BALF bacteria culture was also negative. Cases were not included if they had chronic lung disease, immunodeficiency, bronchopulmonary malformation, or co-infection.

**Figure 1:**
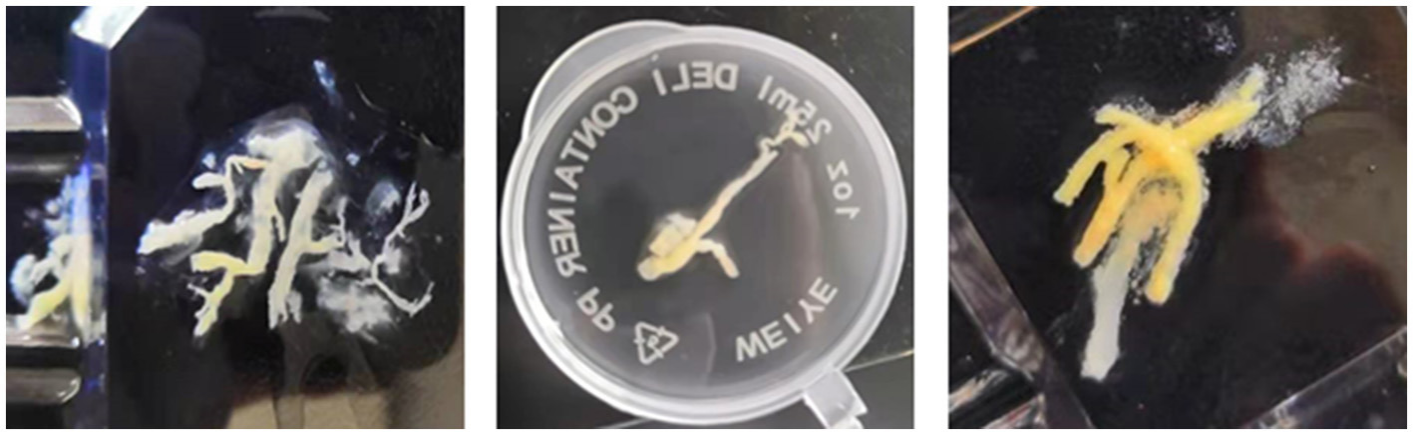
Bronchoscopic findings of a plastic bronchitis patient with mucus plug formation.

Among these 70 children with MPP, 30 were diagnosed with MPP with plastic bronchitis and 40 with non-plastic bronchitis. The MPP children with plastic bronchitis were defined as under bronchoscopy where secretions could be seen to block the lumen and were confirmed by removing branching casts at first-time bronchoscopy (Figure 1). The cohesive and branching casts which filled the airways were removed by lavage, suction, and biopsy forceps. Other children who had blocked lumens without plastic secretions were defined as MPP with non-plastic bronchitis.

A total of 18 children with bronchial foreign bodies who underwent foreign body removal in our hospital at the same time were selected as the control group. The inclusion criteria of the control group were: (1) a clear history of foreign body inhalation and a history of an irritating cough; (2) no inflammatory changes suggested by chest radiographs; (3) no respiratory tract infection within 32 months; and (4) no history of application of hormones and immunosuppressive agents.

The study was approved by the Ethics Committee of the Children's Hospital of Soochow University (2019LW014). Informed consent was obtained from parents or guardians of the children. And the data from patients were analyzed anonymously.

In both MPP or control groups, the information of age and sex was needed. However, clinical data including laboratory test data, the duration of hospital stay and fever, fever peak, lavage times, pleural effusion, atelectasis, and anhelation upon hospital admission and at patient discharge were only collected in MPP children (Table 1). From MPP subjects, peripheral blood samples were obtained within 24 h of admission and used to measure the complete blood count, C-reactive protein (CRP), fibrinogen (Fib), immunoglobulin (IgA/IgG/IgM), subpopulations of lymphocytes, lactate dehydrogenase (LDH), alanine transaminase (ALT), and specific antibody to *M. pneumoniae* and other tests (Tables 1, 2).

**Table 1:** Demographic data and clinical and laboratory characteristics of MPP children with plastic bronchitis and non-plastic bronchitis.

**Clinical parameters**	**Con (*n* = 18)**	**MPP (*n* = 70)**	***P***	**MPP without PB (*n* = 30)**	**MPP with PB (*n* = 40)**	***P***
Age, years	2.1 (1.9, 5.4)	5.0 (3.2, 6.2)	0.052	4.3 ± 2.1	5.2 ± 1.7	0.064
Male, *n*%	12, 66.7%	39, 55.7%	0.404	23, 57.5%	16, 53.3%	0.728
WBC, 10^9^/L	NA	8.1 ± 3.0		7.9 ± 2.7	9.0 ± 3.3	0.121
PLT, 109/L	NA	306.1 ± 105.4		305.9 ± 107.0	318.1 ± 101.0	0.649
CRP, mg/L	NA	9.5 (2.7, 22.2)		5.6 (1.4, 17.3)	20.9 (6.0, 36.5)	<0.001
Fib, g/L	NA	4.0 ± 0.8		4.0 (3.4, 4.5)	4.2 (3.5, 4.8)	0.426
IgA, g/L	NA	1.1 ± 0.6		1.0 ± 0.5	1.2 ± 0.6	0.064
IgG, g/L	NA	8.5 ± 2.2		8.6 ± 2.3	8.3 ± 2.5	0.663
IgM, g/L	NA	1.4 (1.1, 2.1)		1.4 (1.0, 2.1)	1.4 (1.1,1.8)	0.817
CD3, %	NA	62.9 (58.4, 72.1)		63.2 ± 10.7	62.2 ± 12.7	0.749
CD3CD4, %	NA	34.1 ± 9.5		34.7 ± 8.4	32.8 ± 10.1	0.413
CD3CD8, %	NA	26.6 ± 8.3		25.5 ± 8.4	27.9 ± 8.1	0.268
CD3CD (15+56), %	NA	10.4 ± 6.0		10.4 (6.1, 13.3)	7.4 (5.1, 14.3)	0.389
CD3CD19, %	NA	25.7 ± 12.3		24.7 (18.0, 28.9)	23.2 (16.4, 35.6)	0.578
CD3CD8, %	NA	25.9 (20.9, 33.8)		1.4 (1.0,1.9)	1.2 (0.9, 1.5)	0.076
LDH, U/L	NA	420.9 (319.1, 505.6)		340.2 (300.6, 435.4)	479.6 (409.2, 639.3)	<0.001
ALT, U/L	NA	14.9 (20.0, 20.5)		13.5 (10.0, 17.6)	17.4 (10.4, 29.6)	0.068
Duration of fever, days	NA	7.3 ± 3.1		4.8 ± 3.3	9.3 ± 3.2	<0.001
Fever peak	NA	39.6 (39.1, 40.0)		39.4 (39.0, 40.0)	40.0 (39.4, 40.1)	0.004
Hospital stays, days	NA	9.0 (7.0, 12.0)		7.5 (6.0, 10.0)	11.0 (8.0, 13.3)	<0.001
Lavage times, >1, *n*%	NA	28, 40.0%		3, 7.5%	25, 83.3%	<0.001
Pleural effusion, *n*%	NA	17, 24.3%		4, 10.0%	13, 43.3%	0.001
Atelectasis, *n*%	NA	21, 30%		7, 17.5%	14, 46.7%	0.008
Anhelation, *n*%	NA	6, 8.6%		1, 2.5%	5, 16.7%	0.036

*MMP, Mycoplasma pneumoniae pneumonia; PB, plastic bronchitis; WBC, white blood cells; PLT, Platelets; CRP, C-reactive protein; Fib, fibrinogen; IgA/G/M, immunoglobulin A/G/M; CD, cluster of differentiation; LDH, lactate dehydrogenase; ALT, Alanine transaminase*.

**Table 2:** Correlations between expression of MUC5AC, MUC5B, layilin, and clinical parameters.

	**MUC5AC**		**MUC5B**		**Layilin**	
**Clinical parameters**	***r***	***P***	***r***	***P***	***r***	***P***
WBC, 10^9^/L	−0.23	0.221	−0.093	0.626	−0.153	0.42
PLT, 109/L	−0.158	0.442	−0.035	0.867	−0.162	0.429
CRP, mg/L	0.412	0.024	0.441	0.015	0.628	<0.001
Fib, g/L	<0.001	0.999	−0.017	0.928	0.107	0.575
IgA, g/L	0.366	0.047	0.222	0.239	0.175	0.355
IgG, g/L	−0.082	0.668	−0.029	0.88	−0.075	0.692
IgM, g/L	−0.215	0.253	−0.066	0.729	−0.194	0.303
CD3, %	0.294	0.137	0.452	0.018	0.132	0.512
CD3CD4, %	0.111	0.582	0.128	0.526	−0.01	0.961
CD3CD8, %	0.381	0.05	0.543	0.003	0.315	0.109
CD3CD (15 + 56), %	−0.259	0.192	−0.392	0.043	−0.216	0.278
CD3CD19, %	−0.208	0.299	−0.314	0.111	−0.11	0.585
CD3CD8, %	−0.185	0.356	−0.208	0.298	−0.232	0.244
LDH, U/L	0.506	0.004	0.574	0.001	0.483	0.007
ALT, U/L	0.002	0.992	0.16	0.397	0.088	0.644
Duration of fever, days	0.513	0.004	0.596	0.001	0.432	0.019
Fever peak	0.362	0.049	0.217	0.25	0.321	0.083
Hospital stay, days	0.511	0.004	0.476	0.008	0.442	0.014

### Protein Chip

Peripheral blood samples from the GMPP (general *Mycoplasma pneumoniae* pneumonia), refractory *Mycoplasma pneumoniae* pneumonia (RMPP), and control groups were collected. We separately selected three children's serum samples from the groups of healthy, GMPP, and RMPP patients. The criterion of GMPP and RMPP was used as previously described (Li et al., 2019). Protein was isolated and extracted from supernatants of peripheral blood samples using a kit for total protein. Protein concentration was determined using the BCA Protein Assay Kit. According to the producer, the process included these steps: pretreatment of slides, spotting of antigens, washing and blocking, hybridization, scanning of the microarray, and analysis of results.

### Real-Time Fluorescent Quantitation PCR for *M. pneumoniae* in NPA and BALF

Samples (NPA/BALF) were centrifuged and RNAiso Plus was used for nucleic acid extraction from NPA and BALF according to the procedure of PCR for M. pneumoniae. The primers of *M. pneumoniae* were as follows: forward: 5′GCAAGGGTTCGTTATTTG3′; reverse: 5′CGCCTGCGCTTGCTTTAC-3′. The detailed protocol about RNA extraction and procedure of PCR for *M. pneumoniae* 16S rRNA detection was performed as described previously (Ding et al., 2018; Li et al., 2019).

### Detection of MUC5AC, MUC5B, and Layilin by ELISA

According to pathogenetic conditions, BALF was acquired through flexible fiber optic bronchoscopy (Wang et al., 2017). The BALF was collected and centrifuged for detection of *M. pneumoniae* DNA, MUC5AC, MUC5B, and layilin. Commercial ELISA kits were used to detect the expression of MUC5AC, MUC5B, and layilin in the supernatants of BALF. MUC5AC (BP-E15089h), MUC5B (BP-E15086h), and layilin (BP-E15370h) ELISA kits were purchased from Hushang Technology Co. Ltd., Shanghai. All ELISA kits operated in accordance with the manufacturer's instructions.

### Construction of Recombinant Community-Acquired Respiratory Distress Syndrome Toxin

From the NCBI database, we obtained the full-length gene sequence and protein sequence of MPN372. TGA was used to encode tryptophan in M. pneumoniae and TGA was a termination codon in most other species. Eight codon TGAs encoding tryptophan were mutated into TGG. The optimized MPN372 gene sequence was cloned into the pFastBac donor plasmid vector for virus packaging. High-Five cells in the logarithmic growth phase were infected with high titer recombinant virus to express the target protein, which was further purified by a nickel column.

### Construction of Lipid-Associated Membrane Proteins From *M. pneumoniae*

M. pneumoniae strain M129 was purchased from the Institute of Pathogen Biology (Medical College of University of South China). M. pneumoniae was grown in SP4 broth for 72 h at 37°C, centrifuged at 10,000 × g for 20 min, resuspended in saline to yield 1 × 108 CFU/50 μl, and frozen at −80°C until extraction of LAMPs. For detailed experimental procedures, please refer to our previous research (Ding et al., 2018).

### A549 Cell Stimulation by CARDS Toxin or LAMPs *in vitro*

A549 cells were gifted by Professor Jinping Zhang (Soochow University, China). A549 cells were cultured in MEM medium, supplemented with 10% heat-inactivated FCS, 2 mM L-glutamine, 100 U/mL penicillin, and 100 μg/mL streptomycin, at 37°C under 5% CO_2_. The cell density was adjusted to 2 × 10^5^ cells/ml in 12-well plates. The cells were then cultured to the concentration of 5/10/20/30 μg/ml CARDS toxin for 48 h or the cells were then cultured to the concentration of 0/2/6/8/10 μg/ml LAMPs for 16 h. The cells were harvested and immediately stored at −80°C until further analysis.

### MUC5AC, MUC5B, Layilin, IL-6, TNF-α, and IL-1β Detection by Real-Time PCR

The expression of MUC5AC, MUC5B, layilin, IL-6, TNF-α, and IL-1β in the A549 cells which were stimulated by CARDS toxin or LAMPs was measured using real-time PCR. Total RNA was isolated from cells using Trizol (Invitrogen, Carlsbad, CA, USA) reagent and subject to quantitative PCR analysis to measure the expression of mRNA. The Eppendorf Real-Time PCR system was used for quantitative PCR. The PCR conditions were as follows: 40 cycles of 95°C for 5 min, 95°C for 30 s, 60°C for 25 s, and 60°C for 20 s. Data were determined by normalization of expression of β-actin in each sample and presented as means ± SEM. Gene-specific primer sequences are listed in Table 3.

**Table 3:** List of primers used in experiments.

**Gene**	**Primer**
Human-GAPDH	F: CATGTACGTTGCTATCCAGGC
	R: CTCCTTAATGTCACGCACGAT
Human-MUC5AC	F: TGTGGCGGGAAAGACAGC
	R: CCTTCCTATGGCTTAGCTTCAGC
Human-MUC5B	F: AGTTTCCGTCCTTGTCGTAGC
	R: CTGCCCCTTGTTCTGTGACTT
Human-layilin	F: CACAGCCTGCCAGGACCTTTA
	R: TGCACCGGTCATCATTCCA
Human-IL-6	F: ACTCACCTCTTCAGAACGAATTG
	R: CCATCTTTGGAAGGTTCAGGTTG
Human-TNFα	F: CTAATGGTGGAAACCCACAACG
Human-IL-1β	R: TATCGCCAGGAATTGTTGCTG
	F: ACAGATGAAGTGCTCCTTCCA
	R: GTCGGAGATTCGTAGCTGGAT

### Data Analysis and Statistical Analysis

All data were analyzed by SPSS for windows. Categorical data were determined using the Chi-square test. Continuous variables that had a normal distribution were analyzed using Student's *t*-test or the Mann Whitney *U*-test. Statistical significance was determined using one-way ANOVA. Correlations between layilin and MUC5AC or MUC5B were evaluated by Spearman's rank correlation. Figures were generated using GraphPad Prism software and SPSS.

## Results

### The Protein Expression of Layilin Is Altered in the Groups of Children Diagnosed With GMPP and RMPP

**Figure 2:**
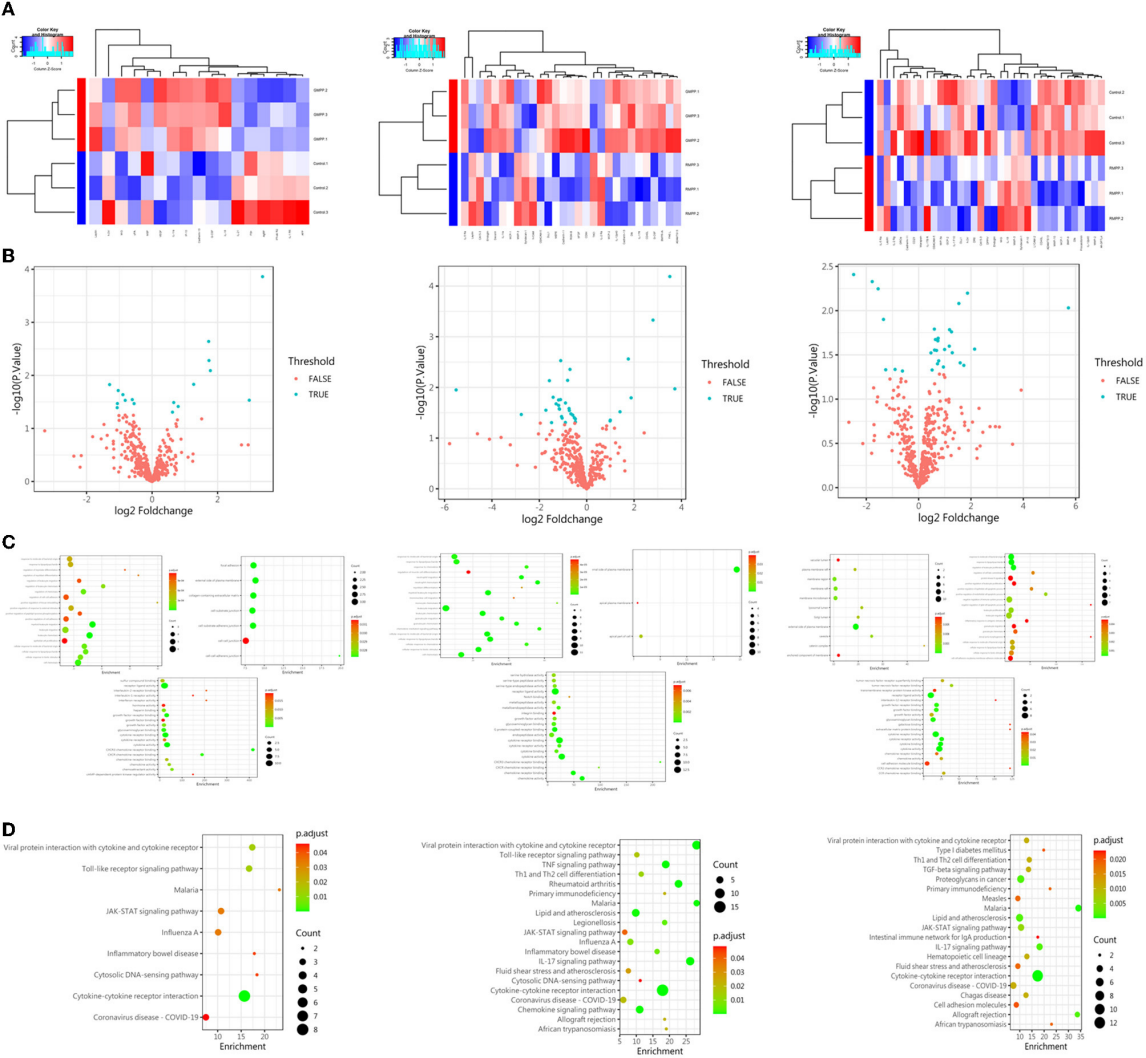
The protein expression of layilin had the most significant difference in each group. **(A)** Heatmap of protein expression abundances among the groups of control, GMPP, and RMPP patients. **(B)** Volcano plot of significantly differentially expressed proteins (*P* < 0.05; FALSE: *P* > 0.05, TRUE: *P* < 0.05) (GMPP vs. CON: *n* = 17; 8 downregulated, 9 upregulated; RMPP vs. CON: *n* = 34; 26 downregulated, 8 upregulated; RMPP vs. GPMM: *n* = 29; 7 downregulated, 22 upregulated). **(C)** Scatter plot of differential protein expression among the groups of control, GMPP, and RMPP patients. Each dot represents one protein. **(D)** KEGG analysis of proteins found enrichment in the cytokine-cytokine receptor interaction pathway among the groups of control, GMPP, and RMPP patients. GMPP, general *Mycoplasma pneumoniae* pneumonia; RMPP, refractory *Mycoplasma pneumonia*e pneumonia.

In order to identify differential protein expression on *Mycoplasma pneumoniae* infection cases, we selected three children's serum samples from the groups of healthy, general *Mycoplasma pneumoniae* pneumonia (GMPP), and refractory *Mycoplasma pneumonia*e pneumonia (RMPP) patients, then analyzed the results of the protein chip. Focusing on the molecules that were distinctively expressed, the level of layilin changed among the three groups (Figure 2A). And we also found that no matter which groups were compared, the expression of layilin was significantly different (*P* < 0.05; Figures 2A–C). Then we used KEGG analysis to predict which signal pathways would involve layilin in the pathogenesis of GMPP or RMPP. Protein enrichment showed that the cytokine-cytokine receptor interaction pathway played an important role and had a more obvious difference then other pathways (*P* < 0.01; Figure 2D). Previous research had demonstrated secretion of inflammatory factors from chondrocytes by layilin signaling in rheumatoid arthritis (RA), they also found that TNF-α upregulated expression levels of layilin in the chondrocytes. However, our team research found TNF-α also had a higher expression in RMPP cases (Li et al., 2019). All of the analyses suggested that layilin may play a role after *Mycoplasma pneumoniae* infection.

### Demographic Data and Clinical and Laboratory Characteristics of MPP Children With Plastic Bronchitis and Non-Plastic Bronchitis

As described in Table 1, there was no significant difference in mean age and gender between control and MPP children. C-reactive protein and LDH in serum were significantly higher in MPP children with plastic bronchitis compared with non-plastic bronchitis cases. Other laboratory test data had no significant difference (Table 1). The occurrences of lavage times, pleural effusion, atelectasis, and anhelation were more frequent in MPP children with plastic bronchitis compared with non-plastic bronchitis cases (all *P* < 0.05). Furthermore, the fever peak was higher in MPP children with plastic bronchitis than non-plastic bronchitis cases. MPP children with plastic bronchitis had longer hospital stays and duration of fever than children with non-plastic bronchitis (*P* < 0.05). The clinical data for MPP children with plastic bronchitis and non-plastic bronchitis are shown in Table 1.

### High Expression of MUC5AC, MUC5B, and Layilin in BALF Between MPP Children With Plastic Bronchitis and Non-Plastic Bronchitis

**Figure 3:**
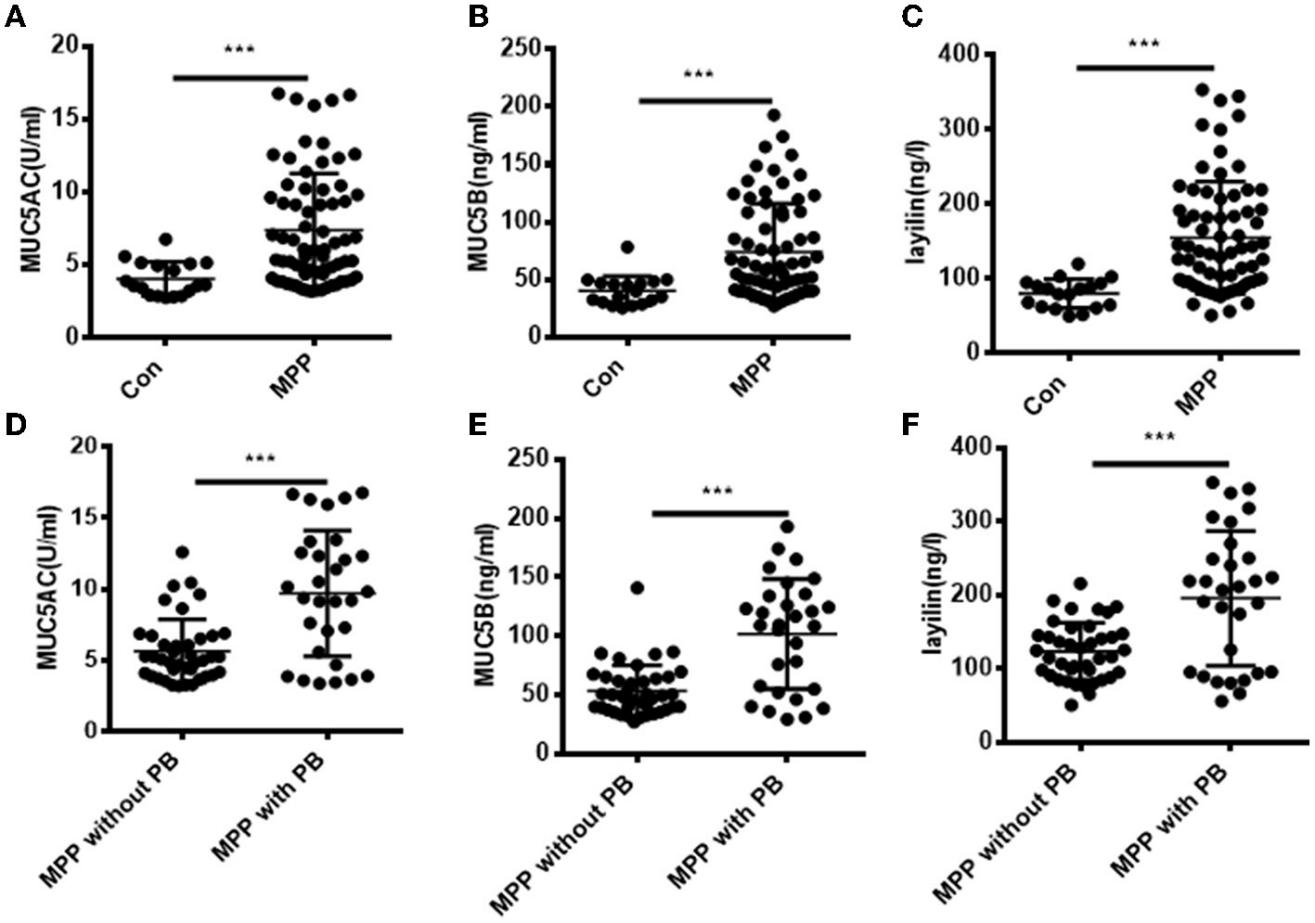
Comparison of MUC5AC, MUC5B, and layilin expression among healthy, MPP, or MPP with PB cases. **(A–C)** The expression of MUC5AC, MUC5B, and layilin in bronchoalveolar lavage fluid (BALF) in the groups of healthy children and MPP cases was measured. ***P < 0.001. **(D–F)** The expression of MUC5AC, MUC5B, and layilin in bronchoalveolar lavage fluid (BALF) in the groups of MPP without plastic bronchitis and MPP with plastic bronchitis was measured. Children with MPP were divided into MPP with plastic bronchitis (*N* = 30) and MPP without plastic bronchitis (*N* = 40). Con, healthy children; MMP, *Mycoplasma pneumoniae* pneumonia; PB, plastic bronchitis. ***P < 0.001.

Previous data (Figure 2) demonstrated that the expression of layilin had a significant difference when comparing any group. Firstly, to explore the level of MUC5AC, MUC5B, and layilin between control and MPP patients, we measured them using ELISA. The results showed that MUC5AC, MUC5B, and layilin were higher in MPP patients (Figures 3A–C). Secondly, to further determine whether patients who had plastic bronchitis could influence the expression of MUC5AC, MUC5B, and layilin, an ELISA was performed to test it. It was shown that the levels of MUC5AC, MUC5B, and layilin significantly increased (Figures 3D–F). All above data suggested that MUC5AC, MUC5B, and layilin may play essential roles in the development of MPP with plastic bronchitis.

### Correlation Between the Levels of MUC5AC, MUC5B, Layilin, and Clinical Parameters in MPP Children With Plastic Bronchitis

**Figure 4:**
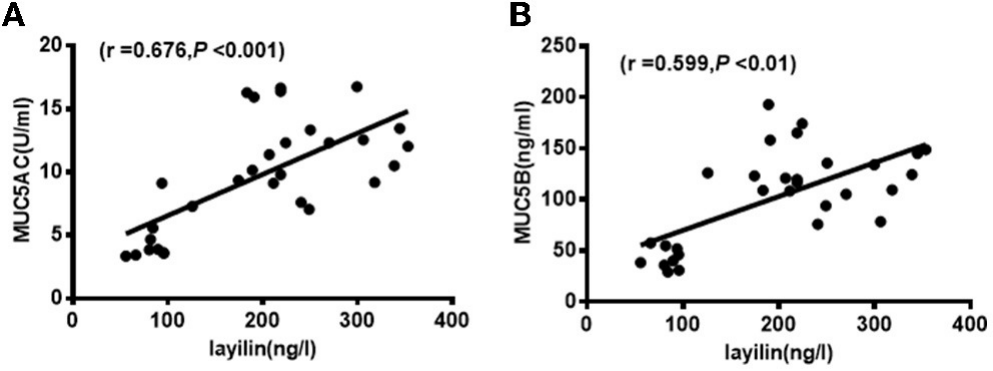
Layilin was positively correlated with MUC5AC and MUC5B level in BALF. **(A)** The positive correlation between layilin and MUC5AC in BALF from MPP children with plastic bronchitis. **(B)** The positive correlation between layilin and MUC5B in BALF from MPP children with plastic bronchitis.

To identify the correlation between the levels of MUC5AC, MUC5B, layilin, and clinical parameters in MPP children with plastic bronchitis, we analyzed data by Spearman's rank correlation. As shown in Table 2, the level of MUC5AC was positively correlated with CRP, IgA, LDH, fever peak, duration of fever or hospital stay, and lavage time (*P* < 0.05). The level of MUC5B was positively correlated with CRP, CD3, CD3CD8, CD3CD (15 + 56), LDH, duration of fever, hospital stay, and lavage time (*P* < 0.05). The level of layilin was positively correlated with CRP, LDH, duration of fever or hospital stay, and lavage time (*P* < 0.05). Furthermore, we analyzed the correlation between layilin and MUC5AC/MUC5B, and found that the level of layilin had a positive correlation with both MUC5AC and MUC5B (Figures 4A,B). Thus, the level of MUC5AC, MUC5B, and layilin may be relevant to the severity of illness.

### Diagnostic Values of MUC5AC, MUC5B, and Layilin in MPP Children With Plastic Bronchitis

**Figure 5:**
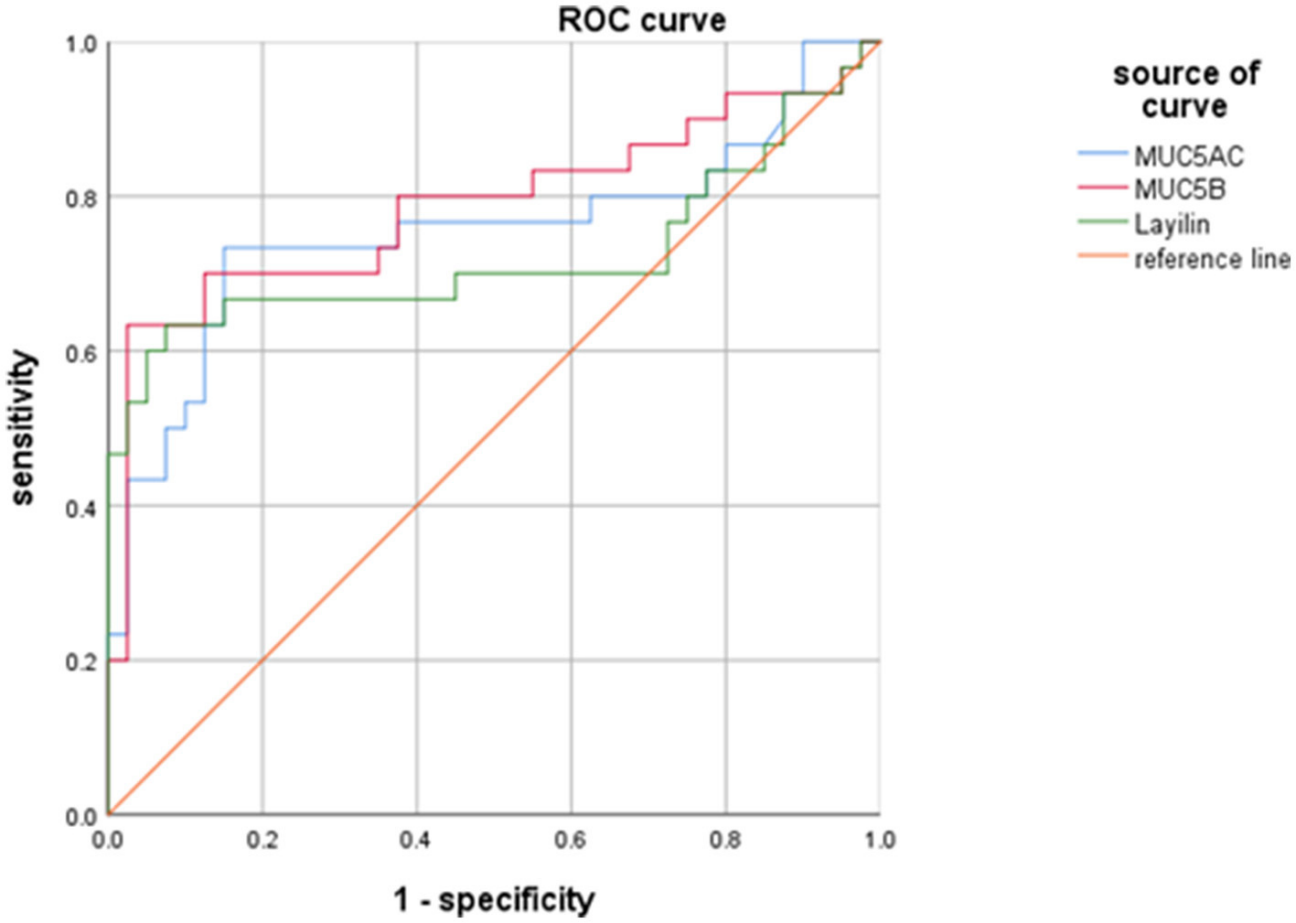
Diagnostic values of MUC5AC, MUC5B, and layilin in MPP children with plastic bronchitis. The cut-off of MUC5AC, MUC5B, and layilin was 7.0 U/ml, 90.3 ng/ml, and 182.7 ng/ml. The AUC of MUC5AC, MUC5B, and layilin was 0.755, 0.787, and 0.723.

To estimate the diagnostic abilities of MUC5AC, MUC5B, and layilin in MPP children with plastic bronchitis, a receiver operating characteristic (ROC) analysis was performed. As shown in Figure 5, MUC5AC, MUC5B, and layilin had similar diagnostic ability for differentiating MPP with plastic bronchitis. The cut-off of MUC5AC, MUC5B, and layilin was 7.0 U/ml, 90.3 ng/ml, and 182.7 ng/ml. The AUC of MUC5AC, MUC5B, and layilin was 0.755, 0.787, and 0.723. Therefore, MUC5AC, MUC5B, and layilin have similar diagnostic ability for MPP children with plastic bronchitis.

### Expression of MUC5AC, MUC5B, Layilin, IL-6, and TNF-α in A549 Cells Stimulated by CARDS Toxin or LAMPs

**Figure 6:**
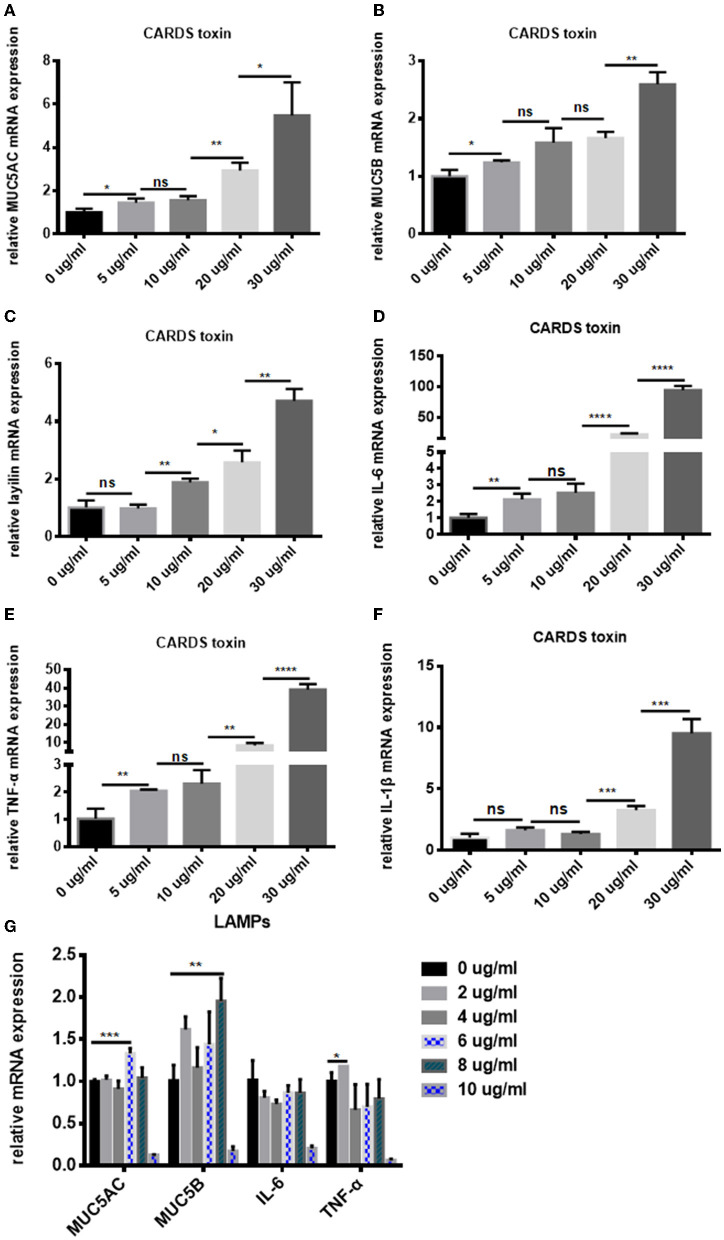
CARDS toxin or LAMPs induced A549 cells to secrete MUC5AC, MUC5B, layilin, IL-6, TNF-α, and IL-1β in A549 cells. Real-time PCR to detect the **(A)** MUC5AC, **(B)** MUC5B, **(C)** layilin, **(D)** IL-6, **(E)** TNF-α, and **(F)** IL-1β mRNA levels in A549 cells which were co-cultured with different concentrations of CARDS toxin (0, 5, 10, 20, and 30 μg/mL) for 48 h. **(G)** Real-time PCR to detect the MUC5AC, MUC5B, IL-6, and TNF-α mRNA levels in A549 cells which were co-cultured with different concentrations of LAMPs (0, 2, 4, 6,8 and 10 μg/mL) for 16 h. NS, no significance; *P < 0.05, **P < 0.01, ***P < 0.001, ****P < 0.0001.

The levels of MUC5AC, MUC5B, layilin , IL-6, TNF-α, and IL-1β in the A549 cells which were stimulated by different concentrations of CARDS toxin or LAMPs were measured using real-time PCR. Data revealed that the levels of MUC5AC, MUC5B, and layilin were increased by CARDS toxin in a dose-dependent manner between 0-30 ug/ml, particularly in the concentration at 30 ug/ml (*P* < 0.05; Figures 6A–C). IL-6, TNF-α, and IL-1β expression was also significantly increased by CARDS toxin stimulated in the concentration at 30 ug/ml (Figures 6D–F). When A549 cells were stimulated by LAMPs, the levels of MUC5AC and MUC5B were not increased in a concentration-dependent manner between 0 and 10 ug/ml, but at a 6 ug/ml concentration of LAMPs stimulation, the level of MUC5AC significantly increased. As shown in Figure 6G, the expression of MUC5B significantly increased at a 8 ug/ml concentration. While the expression of IL-6 decreased by the stimulation of LAMPs. At a 2 ug/ml concentration of LAMPs stimulation, levels of TNF-α significantly increased. These data confirmed that with the increase of CARDS toxin or LAMPs concentration, MUC5AC, MUC5B, and TNF-α mRNA levels in A549 cells were notably upregulated. The results were consistent with the clinical data of early *Mycoplasma pneumoniae* infection. Combined with *in vitro* data and *in vivo* data, it demonstrated that MUC5AC, MUC5B, and layilin play an essential role for prediction in the development of plastic bronchitis caused by MPP.

## Discussion

Plastic bronchitis refers to the local or widespread obstruction of the bronchus by an endogenic foreign body, which results in partial or total ventilation dysfunction of the lung. Plastic bronchitis is generally believed to be related to some bronchial and pulmonary diseases, such as bronchial asthma, bronchiectasis, and other diseases secondary to respiratory infections caused by bacteria, viruses, mycoplasma, and fungi (Efrati et al., 2009; Huang et al., 2019). It has been verified that respiratory infections lead to excessive secretion of airway mucus, and the sticky secretions cannot be effectively removed, which may be an important mechanism of pulmonary diseases accompanied by plastic bronchitis (Eberlein et al., 2008). While the specific pathogenesis of MPP complicated with plastic bronchitis is not fully understood.

In our study, we used a protein chip to identify differential protein expression in *Mycoplasma pneumoniae* infection cases. The level of layilin was distinctively expressed among the three groups and the expression of layilin was significantly different no matter which two groups were compared. KEGG analysis showed that layilin was involved in the pathogenesis of GMPP or RMPP. Protein enrichment analysis also showed that the cytokine-cytokine receptor interaction pathway played an important role and had a more obvious difference than others pathways. Layilin signaling could regulate inflammatory factors in rheumatoid arthritis. Combined with our data, we hypothesized that layilin could, through the cytokine-cytokine receptor interaction pathway, participate in the pathogenesis of GMPP or RMPP. Previous research demonstrated that TNF-α upregulated the expression levels of layilin in the chondrocytes (Asano et al., 2014). Meanwhile, our team found that TNF-α also had a higher expression in RMPP cases (Li et al., 2019). All of the analysis suggested that layilin may play a role after *Mycoplasma pneumoniae* infection. Recently, mucin plugs were the high point in the progress of RMPP. However, MUC5AC and MUC5B played an important role in the formation of mucin plugs (Welsh et al., 2017). Therefore, it is extremely important to understand the role of layilin, MUC5AC, and MUC5B in MPP.

In our research, we found that laboratory test data like CRP, LDH, and the fever peak were significant higher in the group of plastic bronchitis infected by *Mycoplasma pneumoniae*. We also observed that the occurrences of lavage times, pleural effusion, atelectasis, and anhelation were more frequent in MPP children with plastic bronchitis. MPP children with plastic bronchitis had longer hospital stays and duration of fever than children with non-plastic bronchitis. Through a cohort of MP infection patients who underwent bronchoscopy intervention, Xu et al. (2020) found that MPP children with bronchial mucus plugs had longer duration of fever, higher neutrophil percentage, higher CRP level, and higher IL-6 levels compared with those without mucus plugs. Previous studies had shown that CRP, LDH levels, and fever times are related risk factors for *Mycoplasma pneumoniae* pneumonia complicated with plastic bronchitis (Xu et al., 2017). And longer hospital stays, duration of fever, or other symptoms are obviously observed in MPP or PB children (Zheng et al., 2020; Zhang et al., 2021). In summary of previous studies and our study, it was demonstrated that *Mycoplasma pneumoniae* infection combined with plastic bronchitis can significantly aggravate the clinical symptoms of children and the presence of mucus plugs resulted in poor clinical prognosis in children with MPP. Therefore, it is essential to clarify the pathogenesis of MPP complicated with plastic bronchitis.

Mucus plays a vital role in protecting the lungs from environmental factors, but conversely, in some respiratory infection diseases, mucus accumulates in the airways causing obstructions which result in infection and inflammation, and mucus becomes pathologic (Ridley and Thornton, 2018). Previous studies showed that both MUC5AC and MUC5B are increased in COPD sputum (Kirkham et al., 2002). Evans et al. (2015), through a mice model, verified that overproduction of MUC5AC is an effector of allergic inflammation, which participated in the mechanisms of airway mucus plugging and was strongly associated with the development of asthma. Mucus hypersecretion with elevated MUC5B mucin production is a pathologic feature in many airway diseases associated with oxidative stress. It was reported that elevated concentrations of MUC5B leads to mucociliary clearance dysfunction and enhances lung fibrosis in mice (Hancock et al., 2018). Roy et al. (2014), through MUC5B deficient or transgenic mice, found that MUC5B variants may regulate airway homeostasis, disease pathogenesis, and mucosal immune function in humans broadly. In this study, a significant increase in MUC5AC and MUC5B expression from serum was observed in both MPP cases compared with control cases. Furthermore, MUC5AC and MUC5B were significantly higher in serum from children with MPP companied by plastic bronchitis than that in children with MPP without plastic bronchitis. Our data reinforce the pathogenic role of MUC5AC and MUC5B in MPP, especially in MPP with plastic bronchitis. Next, we observed the correlation between the expression of MUC5AC, MUC5B, and clinical parameters in MPP children with plastic bronchitis. In our study, MUC5AC was positively correlated with CRP, IgA, LDH, fever peak, duration of fever, or hospital stay and lavage time. MUC5B was positively correlated with CRP, CD3, CD3CD8, CD3CD (15 + 56), LDH, duration of fever or hospital stay, and lavage time. As mentioned earlier, all those clinical parameters had significant differences in MPP, RMPP, or MPP with plastic bronchitis(Xu et al., 2017; Ding et al., 2018; Li et al., 2019; Zheng et al., 2020; Zhang et al., 2021). Therefore, the clinical parameters and the expression of MUC5AC and MUC5B had suggestive importance to the severity of disease.

A wide array of studies confirms that inflammatory responses play important roles in MPP or plastic bronchitis (Chaudhry et al., 2016; Shimizu, 2016; Yuan et al., 2020; Kim et al., 2021). Layilin, as a member of the hyaluronan (HA) receptors, is widely studied in tumors. To our knowledge, there are almost no articles reporting that layilin is associated with *M. pneumoniae* infection or plastic bronchitis. However, Adachi et al. (2015) found that administration of TNF-α increased the expression of layilin in renal tubular epithelia in mice, and layilin may play roles in the generation of renal interstitial fibrosis in GN *via* TNF-α-induced EMT (Asano et al., 2014). Our team's previous research verified that TNF-α was significantly high in RMPP children, which is a good predictor for refractory *Mycoplasma pneumoniae* pneumonia (Li et al., 2019). Furthermore, layilin is speculated to exacerbate inflammation in rheumatoid arthritis (RA) (Murata et al., 2013). All those studies indicated that layilin plays pathological roles. In this study, we used a protein chip to find that layilin had a significant difference among the groups of healthy, GMPP, and **RMPP** children. Meanwhile, we used an ELISA to detect the expression of layilin, then found that children with MPP had higher layilin levels than healthy children. Importantly, layilin was significantly higher in serum from children with MPP with plastic bronchitis than that in children with MPP without plastic bronchitis.

Pan et al. (2019), through a lung cancer model, found that the function of layilin is involved in lymphatic metastasis, adhesion, and migration. Yu et al. (2011) also confirmed that CD44 plays a role in the pathogenesis of mucus hypersecretion. Casalino-Matsuda et al. (2006) previously showed that ROS-induced MUC5AC expression in NHBE cells is dependent on HA depolymerization and epidermal growth factor receptor (EGFR)/mitogen-activated protein kinase (MAPK) activation. In addition, they also provide evidence that hyaluronan fragments are sufficient to induce CD44/EGFR interaction and downstream signaling that result in MUC5B upregulation, suggesting that hyaluronan depolymerization during inflammatory responses could be directly involved in the induction of mucus hypersecretion (Casalino-Matsuda et al., 2009). Considering that layilin, as a HA receptor, has some similarities to CD44, it may play a role in the pathogenesis of mucus hypersecretion. Therefore, we explored the correlation between layilin and MUC5AC/MUC5B in serum to further confirm whether layilin had an effect on mucus hypersecretion. Here, we observed layilin was positively correlated with MUC5AC and MUC5B from MPP children with plastic bronchitis. Importantly, we evaluated the diagnostic values of layilin, MUC5AC, and MUC5B in MPP children with plastic bronchitis. Our ROC analysis showed that layilin, MUC5AC, and MUC5B had similar diagnostic ability for occurrence of MPP with plastic bronchitis. Altogether, these data indicated that MUC5AC, MUC5B, and layilin had a certain diagnostic value for the occurrence of MPP complicated with plastic bronchitis, and the increase of MUC5AC, MUC5B, and layilin can predict the formation of plastic bronchitis in children with MPP.

Community-acquired respiratory distress syndrome (CARDS) toxin is the only exotoxin produced by *M. pneumoniae* (Su et al., 2021). Kannan et al. (2011) reported that the role of CARDS toxin in infected cells was similar to the cytopathic pathology induced by *M. pneumoniae*, which can independently result in cilia stagnation, vacuolization, nuclear fragmentation, and the release of inflammatory factors. LAMPs were extracted from *Mycoplasma pneumoniae* strains to investigate whether CARDS toxin or LAMPs could induce the expression of MUC5AC, MUC5B, layilin, and inflammatory factors. Data show that recombinant CARDS toxin induced A549 cells to secrete MUC5AC, MUC5B, layilin, IL-6, TNF-α, and IL-1β in a dose-dependent manner caused by a concentration between 0 and 30 ug/ml. Our early findings showed the high expression of MUC5AC, MUC5B, and layilin in BALF from MPP children with or without plastic bronchitis. And our team's previous data indicated that CARDS toxin could induce TNF-α expression and thereby was involved in enhancing lung inflammatory cell infiltration and mucus secretion (Li et al., 2019). These results indicate that CARDS toxin can induce inflammation and mucus secretion. All results indicated that a high concentration of CARDS toxin can cause high secretion of mucus and high release of inflammatory factors. High concentration of LAMPs could stimulate A549 cells to secrete MUC5AC and MUC5B. TNF-α was increased by the stimulation of LAMPs. There are no reports on the expression of MUC5AC and MUC5B and its association with other inflammatory factors in A549 cells after stimulation with LAMPs. A previous study showed that LAMPs could stimulate TNF-α increase (Ding et al., 2018). In the present study, it was demonstrated that LAMPs from *M. pneumoniae* could induce MUC5AC and MUC5B expression. Meanwhile, MUC5AC and MUC5B may induce inflammation and mucus secretion through a TNF-α-associated pathway. This deserves further research. All those results demonstrated that MUC5AC, MUC5B, and layilin were related to *Mycoplasma pneumoniae* infection.

Our data also showed a high expression of MUC5AC, MUC5B, and layilin in BALF from MPP children with plastic bronchitis. While, the level of inflammation factors like IL-6, TNF-α, and IL-1β also increased gradually in a certain range of CARDS toxin concentration. Studies showed that IL-1β significantly suppressed the expression of LAYN in human articular chondrocytes and synoviocytes. TNF-α upregulated expression levels of LAYN in the chondrocytes (Asano et al., 2014). These results indicated the expression of layilin may influence the expression of IL-6, TNF-α, and IL-1β.

In conclusion, the expression of layilin, MUC5AC, and MUC5B was increased in MPP children with plastic bronchitis, which are good diagnostic biomarkers for predicting the formation of plastic bronchitis in children with MPP. The high level of MUC5AC and MUC5B may be induced by layilin. Layilin may, through regulate inflammatory factors, affect the outcome of MPP cases. Further exploration of the specific mechanism is needed. This study provides a new basis for the early recognition and diagnosis of MPP complicated with plastic bronchitis.

## Data Availability Statement

The datasets presented in this study can be found in online repositories. The names of the repository/repositories and accession number(s) can be found in the article/supplementary material.

## Ethics Statement

The studies involving human participants were reviewed and approved by the Ethics Committee of the Children's Hospital of Soochow University (2019LW014). Written informed consent to participate in this study was provided by the participants' legal guardian/next of kin. Written informed consent was obtained from the individual(s), and minor(s)' legal guardian/next of kin, for the publication of any potentially identifiable images or data included in this article.

## Author Contributions

YM performed the experiments, data analysis, and drafted the manuscript. YG participated in collecting data and samples, data analysis, and revised the manuscript. XZ, WG, TW, and HMS collected clinical data, peripheral blood samples, and BALF. YY, YW, MW, HQS, and CH supervised data collection and carried out the initial interpretation of data. ZC and LF participated in data analysis and interpretation. ZC had primary responsibility for the study design, performed experiments, data analysis, interpretation of data, and wrote the manuscript. All authors read and approved the final manuscript.

## Funding

This work was supported by grants from the National Natural Science Foundation of China (Grant Numbers: 81870006, 81900006, and 81970027), Key Lab of Respiratory Disease of Suzhou (Grant Number: SZS201714), Key Laboratory for Diagnosis and Treatment of Immune Diseases in Children (Grant Number: SZS201808), Jiangsu Provincial Medical Youth Talent Project (Grant Number: QNRC2016766), Suzhou Medical Youth Talent Project (Grant Numbers: GSWS2019047 and GSWS2020053), Social Development Projects of Jiangsu Province (Grant Number: BE2019671), Medical Health Science and Technology Project of Yili (Grant Numbers: YZ2021YD029 and YL2020LH03), Suzhou Livelihood Science and Technology Project (Grant Number: SKJY2021104 to TW), and Science and Technology Project of Suzhou City (Grant Number: KJXW2020025 to TW).

## Conflict of Interest

The authors declare that the research was conducted in the absence of any commercial or financial relationships that could be construed as a potential conflict of interest.

## Publisher's Note

All claims expressed in this article are solely those of the authors and do not necessarily represent those of their affiliated organizations, or those of the publisher, the editors and the reviewers. Any product that may be evaluated in this article, or claim that may be made by its manufacturer, is not guaranteed or endorsed by the publisher.
